# Supplementary material for: Natural spatial pattern—When mutual socio-geo distances between cities follow Benford’s law
Source: PLoS One. 2022 Oct 20;17(10):e0276450. doi: 10.1371/journal.pone.0276450 (PMC9584388; doi:10.1371/journal.pone.0276450)
Supplement: Attachment — Submitted filename: Response to reviewers.docx [file pone.0276450.s001.docx]

**Answers to reviewers**

Dear reviewers,

thank you for your valuable comments. We have addressed all points you raised. All the changes were evidenced in track-mode version attached. The table below summarises our answers (with lines references to track-mode version).

Best regards,

Authors

**Reviewer #1**

| **Comment from reviewer** | **Our answer** |
| --- | --- |
| I tend to disagree with the authors that the use of “Benford distribution to analyse the geolocation of cities and their population in the majority of countries” is a pioneering work. Why?  Benford distribution is one of many heavy-tailed distributions such as Zipf’s law, Pareto principle (80/20 principle), Korcak's Law, Horton's laws, Gutenberg–Richter law, Bradford's law (1:n:n^2), Lotka’s law, and Moore’s law.  According to the previous study by B. Jiang and his co-workers, all countries’ cities follow Zipf’s law, with the exponent being around 1.0; see the following studies: <https://www.tandfonline.com/doi/abs/10.1080/13658816.2014.988715?journalCode=tgis20>  Note that natural cities (or all human settlements that are objectively defined) can be defined not only at country level, but also at city level, or the city of the city level. <https://www.tandfonline.com/doi/full/10.1080/13658816.2018.1427754>  It is not surprising to me at all that mutual socio-geo distances between cities follow Benford’s law, since Benford’s law is a much more relaxed law than Zipf’s law. If natural cities follow Zipf’s law, they will surely follow Benford’s law.  The authors are still constrained by the conventional thinking, i.e., population as city size. This conventional thinking is very backward since population is initially and essentially for census or administrative purposes rather than for scientific purposes. With the advance of geospatial big data, we should adopt new ways of thinking. The current state of the art on Zipf’s law is to use natural cities, with which all countries’ natural cities follow Zipf’s law without a single exception. Even for Singapore as a city country whose natural cities can be generated to fit Zipf’s law.  The essence of Benford’s law and Zipf’s law is the notion or recurring notion of far more smalls than larges. This notion has been formulated as the scaling law (<https://link.springer.com/article/10.1007/s10708-014-9537-y> ), which can be manifested by head/tail breaks (<https://en.wikipedia.org/wiki/Head/tail_breaks> ). I therefore suggest the authors use the state of the art concepts (natural cities, the scaling law, and ht-index) to re-examine their findings. | We read with interest your opinions and four recommended papers. They helped us improve the quality of the paper. We build the reference of those papers as an important supplement to the literature review for Zipf and the modern approach to analyse urban data. We agree with all your claims regarding the approach to Zipf. Zipf is the first choice when analysing the urban population. However, we feel that we did not underline well enough the difference in our study.  Therefore we added a phrase in which we explain it – see lines 202-210:  “*Using Zipf in this study has two functions: first, it builds a well-understood reference to other studies dealing with urban populations; second, it is to show the difference between Zipf and Benford. The fundamental difference is that Zipf never refers to precise geo-location of cities of which population is being studied – Zipf is locationally invariant - even if cities with their populations would be relocated, Zipf value will stay the same. Oppositely, Benford in our approach with 3D distance, has important spatial features. To visualise it better, analysis of Zipf is on a vector of population, while Benford's on a complex three-dimensional network of cities and inhabitants, where the reshuffling of urban locations or their populations or both matters for the outcome.*”  We also underlined this in the abstract, by merging with reference to a recommended study on natural cities, where the word “natural” is an important analogy to our research – please see lines 79-90:  “*Population studies mostly use the Zipf law on administrative data (Cristelli et al., 2012; Cottineau, 2017; Cottineau, 2022) – the general conclusion is that this law holds, but the results strongly depend on the methods and data applied. An interesting approach is by using night-light satellite images to address not administrative population data but a natural settlement (non-biased data), which often goes beyond the official contour of the city. Jinag et al. (2015) analyses with Zipf the global urban settlement for “natural cities” and found Zipf conformity in almost 60% of countries. Decker et al. (2007) in contrary for nigh-light data shows lognormal distribution fits data much better than Zipf. However, Zipf law (and lognormal distribution) never considers the geographical location of the cities and does not see cities as a socio-geo network. This simplified, one-dimensional approach neglects spatial components, which seems naturally important if a mass of people concentrates in neighbouring or distant cities..*”  In fact, we understand the word “natural” somehow differently, due to the context of the study.  Please see the lines 256-261:  “*We discover that despite the different location patterns of diversified cities among countries (due to the natural conditions, the shape of the country, population density, history etc.) the urban organisation worldwide follows a* ***natural spatial pattern****. What does it mean? For ages, people, in their decisions where to locate, behaved naturally, thus* ***spatial separation and mass of cities are not random but constitute a complex three-dimensional network conforming with the ordinary course of nature.***”  When expanding the literature review, we added an interesting concept of living structures, together with other modern studies of urban structure – see the lines 90-101:  “*Dziecielski et al. (2021) offer a good review of those studies, from Christaller’s Central Place Theory and New Economic Geography (NEG), through Tinbergen-Bos and Tellier models to Systemic Economic Geography (SEG) focusing on neo-Newtonian approach to see “spheres of influence” and gravity forces of 14 European urban agglomerations by using data on accessibility, cultural interactions, economy, environment, liveability and R&D. Ioannides and Overman (2004) try (and fail) to explain urban system with city location and their socio-economic interactions. Most modern view of geographic space as a living structure explains human activity (like Tweeting) in an interesting way with a topological representation of the built environment and big data (Jiang & Ren, 2019). However, those studies are usually based on socio-economic relations, are selective territorially, consider major cities only, refer to a relatively short time span and are not anchored in history.*”  We have also extended in a few places the text referring to Zipf and Benford:  In lines 189-191:  “*Benford law, together with Zipf law, belong to a power law (e.g. Pietronero et al., 2001). Even if they behave similarly being heavy-tailed distributions (Jiang et al., 2013), they keep different properties (Pietronero et al., 2001).*”  Lines 198-201:  “*Many studies confirm that urban populations follow the Zipf law (e.g. Jiang et al., 2015), but also meta-analysed of Zipf law studies show that there is no single Zipf law (Cottineau, 2017) and Zipf results differ between disciplines and the focus of study (Cottineau, 2022).*”  We have also changed the text on results regarding Zipf - in lines 348-380:  “***Population distribution and Zipf's law conformity*** *– for population data, we checked Zipf's law conformity. We did not restrict data within the countries by setting any threshold for city size. All cities available in the database were used in calculations to keep coherence with the Benford analysis. As we focus on complex city network analysis, we were interested in a possibly comprehensive picture of the urban system. We evidence that only in a few countries (23 out of 169, 14%) Zipf coefficient is around -1 (±0.05, from -0.95 to -1.05), while in a majority (123 out of 169, 73%) the Pareto form Zipf coefficient was between (-0.37, -0.94), and only in few countries (23/169, 14%) its absolute value exceeded -1 (-1.056, -1.411) (Fig.4). This marginal share was already confirmed in the literature by Nota and Song (2012), who analysed what number of cities in a subsample conforms with Zipf – their results show that in the case of the U.S. the Zipf conformity requires between 140 and 205 cities, and for more or less cities it disperses from 1 significantly. Cristelli et al. (2012) also confirm that Zipf holds conditionally in selected subsamples only. Countries with Zipf-like population distribution are diversified: a the number of cities varies from 27 to 985, the coefficient of variation of city size within the country is between 0.84 and 7.4, Clark-Evans R is between 0.42 and 1.25, and countries are on all continents.*  *The correlation between Zipf's coefficient and 3D MAD is average (0.41) - the higher Zipf's coefficient (the more similar / less diversified the cities within the country), the higher MAD (the weaker Benford conformity). However, this relation is not strong, and the information included in Zipf and Benford differs. This is in line with the statistical properties of Benford distribution. In general, statistical distributions which are around the normal or regular pattern and tend to follow symmetry almost never follow Benford (Formann, 2010). Much more Benford-fitting are survival-like distributions, where the frequency of occurrence is an inverse function of the object's size – when smaller objects appear more often than the bigger one and objects are more diversified.*  *This finding is in line with the observation of Nota and Song (2012), who show that in the case of datasets for China, the Zipf coefficient equal 1 is rarely achieved. They explain this with a long-term policy of balancing the size of cities by limiting the growth of big cities, reasonably developing middle-size cities and strongly supporting the growth of small cities. However, in light of the results of this study, policy based on a non-spatial vector of population data lacks the complex network features inherited in 3D Benford approach. Thus, deeper conclusions from this part of the study are not justified.*”  We find as very inspiring the comment on the Paretian way of thinking, this element was also included in the paper,  In intro in lines 102-112:  “*Our study approaches this old problem of urban geography very differently. We treat the geographic system of cities as a complex non-linear three-dimensional network, which is not random or independent but reveals high spatial heterogeneity. We search for hidden order which is to be explained with Benford law. We consider the socio-geographic three-dimensional (3D) space (localisation and population) and its components: two-dimensional geographic distance (2D) and population one-dimensional distance (1D). As Benford law holds in natural datasets, we claim that spatial phenomena like urbanisation, when created naturally, are Benford-like.* *In contrast, spatial processes driven by the other (e.g. political) rules of ordering fail this conformity. In the natural spatial pattern of urban settlement, spatial separation and mass of cities are not random but constitute a complex three-dimensional network conforming with the ordinary course of nature.*”  And in lines 450-467 (newly added discussion section in the empirical section):  “*All those analyses show that both factors, allocation of the population as well as the location of cities, matter for natural distributions, and Benford's conformity is an output of very special complex settings. This confirms the hypothesis that cities in the three-dimensional setting of location and population constitute a complex non-linear, non-random network. We claim that we discover hidden order with Benford law, which explains the observed spatial heterogeneity. This interdependency of human location decisions in space is part of the Paretian way of thinking (Jiang, 2015) and evidences that the assumption of the Gaussian linear world is too simplistic. This finding has a critical implication for the simulation study. As indicated by Jiang (2015), spatial heterogeneity, manifesting in uneven distributions, should be perceived as a mix of concentrations in a human-made built environment. In section 3 we show that, indeed, a mixture of point patterns and distributions may replicate this natural urban geographic system.*  *One should underline that the presented analysis is much more statistical than economic or political. We analyse all world countries, which naturally are at very different stages of development, facing diversified problems, running various policies, and being not uniform culturally or politically. All those factors are neglected, and only the city's location and population are considered. This, of course, limits the range of study but allows for focusing on statistical phenomena and long-term evolutionary processes.*”  We feel that all your comments were based on the assumption (appearing at the beginning of the review), which puts the equal sign between Zipf and Benford. Besides all written above, we underline that the correlation between Zipf and all measures in our studies (MAD for 1D, 2D, 3D distances, R in Clark-Evans test) is low – varies from 0.213 to 0.408 (see Fig.6 first row) and text in lines 420-423:  *“Zipf for population does not bring the same information as Benford for population (corr=0.27 with MAD on population vector and corr=0.4 with MAD on population distance), link remains moderate when location additionally considered (corr=0.41 with MAD on 3D soc-geo distance).”*  and in lines 426-427:  “*Zipf is also loosely linked to spatial patterns of urban locations (RofClarkEvans) (corr=0.21) (in fact Zipf is a-spatial metric).*”  Thus, we feel it is worth studying Benford separately, even if, in some simpler cases, one can, in fact, say that Zipf and Benford are (almost) the same.  Besides the corrections mentioned above, we also included a few other amendments, which resulted from another review as well as our reading of the text. References were also extended. |

**Reviewer #2**

| **Comment from reviewer** | **Our answer** |
| --- | --- |
| This paper applied Benford’s law in a spatial context to examine the geo-location of cities and their population. The authors justify the Benford-like spatial disputation of cities and inhabitants worldwide following the evolutionary process, which is a novel finding. The paper addressed a very important question which has not been comprehensively studied in previous literature. The English writing is acceptable but with minor grammatical errors. Overall, this paper is well-written and the structure of the manuscript is very clear. However, a few issues should be addressed before it can be published: | Thank you for the positive assessment of the paper. |
| The authors mentioned a few studies on location and population of cities such as Dziecielski et al. (2021). However, how these studies approach the issue should be elaborated in detail. I suggest the authors provide more relevant studies to better position the contribution of this paper. | This remark was taken seriously. We have screened deeper into the literature in search of different approaches in studies on urban systems. We have extended a paragraph in the intro to cover more methods – see lines 78-101:  “*In the last hundred years, there were many concepts on how to explain the location and population of cities. Population studies mostly use the Zipf law on administrative data (Cristelli et al., 2012; Cottineau, 2017; Cottineau, 2022) – the general conclusion is that this law holds, but the results strongly depend on the methods and data applied. An interesting approach is by using night-light satellite images to address not administrative population data but a natural settlement (non-biased data), which often goes beyond the official contour of the city. Jinag et al. (2015) analyses with Zipf the global urban settlement for “natural cities” and found Zipf conformity in almost 60% of countries. Decker et al. (2007) in contrary for nigh-light data shows lognormal distribution fits data much better than Zipf. However, Zipf law (and lognormal distribution) never considers the geographical location of the cities and does not see cities as a socio-geo network. This simplified, one-dimensional approach neglects spatial components, which seems naturally important if a mass of people concentrates in neighbouring or distant cities. There are also a few other approaches. Dziecielski et al. (2021) offer a good review of those studies, from Christaller’s Central Place Theory and New Economic Geography (NEG), through Tinbergen-Bos and Tellier models to Systemic Economic Geography (SEG) focusing on neo-Newtonian approach to see “spheres of influence” and gravity forces of 14 European urban agglomerations by using data on accessibility, cultural interactions, economy, environment, liveability and R&D. Ioannides and Overman (2004) try (and fail) to explain urban system with city location and their socio-economic interactions. Most modern view of geographic space as a living structure explains human activity (like Tweeting) in an interesting way with a topological representation of the built environment and big data (Jiang & Ren, 2019). However, those studies are usually based on socio-economic relations, are selective territorially, consider major cities only, refer to a relatively short time span and are not anchored in history.”* |
| The structure of the paper should be revised. For example, the paragraph in line 153 talks about Zipf’s law, which fits better in a later section of result or discussion. The sentence “As our empirical results will show relatively poor Zipf's law conformity” should not be in the methods section. I suggest the authors reorganise the contents so that the flow is more smoothly and readers do not need to go back and forth. | You are right, the structure was corrected.  In section 1 we kept the methodological presentation and discussion of methods used (also with Zipf coefficient) and deleted any references to results – see lines 189-210:  “*Benford law, together with Zipf law, belong to a power law (e.g. Pietronero et al., 2001). Even if they behave similarly being heavy-tailed distributions (Jiang et al., 2013), they keep different properties (Pietronero et al., 2001). Zipf law is traditionally applied to analyse the size of cities within a given country. Zipf's coefficient in the Pareto form is usually calculated as a regression coefficient (slope) from the linear model, in which the dependent variable y is the log of ranks of cities (the biggest city is no 1, the smallest city is the last number), while explanatory variable x is the log of the population of cities. The Lotka form assumes opposite relation, that log of population (y) is explained with long of rank (x) (Cottineau, 2017). Regression coefficients around -1 evidence intra-country Zipf's law conformity. Many studies confirm that urban populations follow the Zipf law (e.g. Jiang et al., 2015), but also meta-analysed of Zipf law studies show that there is no single Zipf law (Cottineau, 2017) and Zipf results differ between disciplines and the focus of study (Cottineau, 2022).*  *Using Zipf in this study has two functions: first, it builds a well-understood reference to other studies dealing with urban populations; second, it is to show the difference between Zipf and Benford. The fundamental difference is that Zipf never refers to precise geo-location of cities of which population is being studied – Zipf is locationally invariant - even if cities with their populations would be relocated, Zipf value will stay the same. Oppositely, Benford in our approach with 3D distance, has important spatial features. To visualise it better, analysis of Zipf is on a vector of population, while Benford's on a complex three-dimensional network of cities and inhabitants, where the reshuffling of urban locations or their populations or both matters for the outcome.*”  Zipf results were discussed in section 2 in the subsection on Zipf. They were better elaborated – see lines 348-380  We considered merging methodological and empirical sections, but it became less transparent and lengthy. |
| The big versus small country of 100 cities is an interesting finding. However, as globalisation puts cities of different countries in close relationships such as trading, migration and change of administrative boundaries, the border effect should be discussed to extend Benford’s law non/conformity. | It is the right remark. We included two comments on that. To be honest, this is an issue for a separate paper.  First is in subsection “sample size” in current lines 298-307:  “*The threshold of 100 cities to distinguish between big and small countries was taken from statistical outcomes (as the bottom part of the box in marginally acceptable conformity). However, it raises an important question of whether globalisation processes, trading, migrations and other interactions impact the analysis. We adopted the institutional perspective, which assumes that resettlement of average inhabitants is much easier within the country than between the countries due to language barriers, formalities, different institutional systems, permissions, certifications, schooling systems, etc. This approach enhances the role of national borders. This spirit of analysis can be confirmed by the study of the US urban system by Ioannides and Overman (2004), who claim that interactions between the cities cannot explain the size of cities (and thus mobility between them). However, this is an issue for further investigations.*”  The second is in subsection “spatial point-patterns” in current lines 329-335:  “*We also find that in countries with less than 100 cities Clark-Evans test detected a predominantly random pattern (43%, 30/70) and ordered pattern (37%, 26/70), while the agglomerated pattern was revealed only in 20% of cases (14/70). As Benford conformity mostly appears in agglomerated patterns, this justifies why Benford conformity was rarely detected in small countries. What remains unpuzzled here is if countries with less than 100 cities indeed do not have clustered patterns, or is it the statistical effect that agglomeration is hard to detect in a small sample.*” |
| The discussion in line 247 can be further expanded at the policy level as opposed to the formation of the natural spatial pattern. | This comment referred to section ***Population distribution and Zipf's law conformity****,* which had just one paragraph. We improved this part. We included, in brief, the policy-oriented discussion in the third paragraph of this fragment. Now it is in lines 348-380:  “***Population distribution and Zipf's law conformity*** *– for population data, we checked Zipf's law conformity. We did not restrict data within the countries by setting any threshold for city size. All cities available in the database were used in calculations to keep coherence with the Benford analysis. As we focus on complex city network analysis, we were interested in a possibly comprehensive picture of the urban system. We evidence that only in a few countries (23 out of 169, 14%) Zipf coefficient is around -1 (±0.05, from -0.95 to -1.05), while in a majority (123 out of 169, 73%) the Pareto form Zipf coefficient was between (-0.37, -0.94), and only in few countries (23/169, 14%) its absolute value exceeded -1 (-1.056, -1.411) (Fig.4). This marginal share was already confirmed in the literature by Nota and Song (2012), who analysed what number of cities in a subsample conforms with Zipf – their results show that in the case of the U.S. the Zipf conformity requires between 140 and 205 cities, and for more or less cities it disperses from 1 significantly. Cristelli et al. (2012) also confirm that Zipf holds conditionally in selected subsamples only. Countries with Zipf-like population distribution are diversified: a the number of cities varies from 27 to 985, the coefficient of variation of city size within the country is between 0.84 and 7.4, Clark-Evans R is between 0.42 and 1.25, and countries are on all continents.*  *The correlation between Zipf's coefficient and 3D MAD is average (0.41) - the higher Zipf's coefficient (the more similar / less diversified the cities within the country), the higher MAD (the weaker Benford conformity). However, this relation is not strong, and the information included in Zipf and Benford differs. This is in line with the statistical properties of Benford distribution. In general, statistical distributions which are around the normal or regular pattern and tend to follow symmetry almost never follow Benford (Formann, 2010). Much more Benford-fitting are survival-like distributions, where the frequency of occurrence is an inverse function of the object's size – when smaller objects appear more often than the bigger one and objects are more diversified.*  *This finding is in line with the observation of Nota and Song (2012), who show that in the case of datasets for China, the Zipf coefficient equal 1 is rarely achieved. They explain this with a long-term policy of balancing the size of cities by limiting the growth of big cities, reasonably developing middle-size cities and strongly supporting the growth of small cities. However, in light of the results of this study, policy based on a non-spatial vector of population data lacks the complex network features inherited in 3D Benford approach. Thus, deeper conclusions from this part of the study are not justified.*” |
| Size or shape of the country. It seems the comparison provided on the four case studies is incomplete. What does the comparison imply? The four countries are at very different development stages, geopolitical locations, and with various economic/population sizes. | You are right, and we appreciate the very different perspective and optics toward the problem. The authors of the paper are economists, and we are pretty aware of the diversity of world countries, especially in terms of growth, technology, politics, trade, etc.  When correcting the paper, we considered including the economic analysis; however, the paper would become a book then. That is a very interesting path, and we are already thinking about another paper dealing with those issues. However, in this paper, we limited ourselves. We added the comment in the newly added section on a discussion of results at the end of the empirical part – see lines 450-467.  ***“Discussion of results***  *All those analyses show that both factors, allocation of the population as well as the location of cities, matter for natural distributions, and Benford's conformity is an output of very special complex settings. This confirms the hypothesis that cities in the three-dimensional setting of location and population constitute a complex non-linear, non-random network. We claim that we discover hidden order with Benford law, which explains the observed spatial heterogeneity. This interdependency of human location decisions in space is part of the Paretian way of thinking (Jiang, 2015) and evidences that the assumption of the Gaussian linear world is too simplistic. This finding has a critical implication for the simulation study. As indicated by Jiang (2015), spatial heterogeneity, manifesting in uneven distributions, should be perceived as a mix of concentrations in a human-made built environment. In section 3 we show that, indeed, a mixture of point patterns and distributions may replicate this natural urban geographic system.*  *One should underline that the presented analysis is much more statistical than economic or political. We analyse all world countries, which naturally are at very different stages of development, facing diversified problems, running various policies, and being not uniform culturally or politically. All those factors are neglected, and only the city's location and population are considered. This, of course, limits the range of study but allows for focusing on statistical phenomena and long-term evolutionary processes.”*  Besides the corrections mentioned above, we also included a few other amendments, which resulted from another review as well as our reading of the text. References were also extended. |
